# Controllable Capillary Assembly of Magnetic Ellipsoidal Janus Particles into Tunable Rings, Chains and Hexagonal Lattices

**DOI:** 10.1002/adma.202006390

**Published:** 2021-01-14

**Authors:** Qingguang Xie, Jens Harting

**Affiliations:** ^1^ Department of Applied Physics Eindhoven University of Technology P.O. Box 513 5600MB Eindhoven The Netherlands; ^2^ Helmholtz Institute Erlangen‐Nürnberg for Renewable Energy (IEK‐11) Forschungszentrum Jülich Fürther Str. 248 90429 Nürnberg Germany; ^3^ Department of Chemical and Biological Engineering and Department of Physics Friedrich‐Alexander‐Universität Erlangen‐Nürnberg Fürther Str. 248 90429 Nürnberg Germany

**Keywords:** controllable assembly, ellipsoids, Janus particles, particle‐laden fluid interfaces

## Abstract

Colloidal assembly at fluid interfaces has a great potential for the bottom‐up fabrication of novel structured materials. However, challenges remain in realizing controllable and tunable assembly of particles into diverse structures. Herein, the capillary assembly of magnetic ellipsoidal Janus particles at a fluid–fluid interface is reported. Depending on their tilt angle, that is, the angle the particle main axis forms with the fluid interface, these particles deform the interface and generate capillary dipoles or hexapoles. Driven by capillary interactions, multiple particles thus assemble into chain‐, hexagonal‐lattice‐, and ring‐like structures, which can be actively controlled by applying an external magnetic field. A field‐strength phase diagram is predicted in which various structures are present as stable states. Owing to the diversity, controllability, and tunability of assembled structures, magnetic ellipsoidal Janus particles at fluid interfaces could therefore serve as versatile building blocks for novel materials.

## Graphical abstract

Magnetic ellipsoidal Janus particles assemble into rings, chains, and hexagonal‐lattice structures at fluid–fluid interfaces driven by capillary interactions, where a dynamical transition between these assembly states can be obtained by dynamically manipulating the external magnetic field.

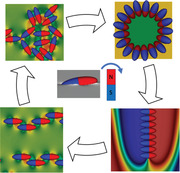

Self‐assembly of particles has received considerable attention with respect to their potential applications in advanced technologies, such as displays, sensors and optoelectronic devices. For certain industrial applications including large‐area coatings, the assembly of particles can prove far more efficient than “top‐down” approaches such as lithography. However, controlling particles into highly tunable and predictable structures remains a challenge.^[^ ^1^, ^2^, ^3^, ^4^ ^]^ Herein, we report a highly controllable and versatile strategy for the fabrication of ordered structures via capillary assembly of magnetic ellipsoidal Janus particles at a fluid–fluid interface.

Colloidal particles strongly attach at a fluid–fluid interface^[^ ^5^ ^]^ and deform the interface due to their weight, anisotropic shape and roughness.^[^ ^6^, ^7^, ^8^, ^9^ ^]^ If neighboring particles generate deformations of the interface that overlap, capillary interactions arise which drive the particles to assemble into ordered structures. Such capillary interactions can be characterized by the modes of interface deformation. In the limit of small interface deformation, the interface height **h** around the particle can be described according to the Young–Laplace equation ∇**h** = 0 and it can be analyzed by a multipole expansion in analogy with electrostatics.^[^ ^8^, ^10^ ^]^ In the case of heavy particles attached to the interface, the interface is pushed down by the particles resulting in a monopolar deformation.^[^ ^6^ ^]^ For particularly small or light particles, the contact line where particle and fluid–fluid interface meet can undulate due to anisotropic shapes or the roughness of the particle surface and, thus, induce quadrupolar or hexapolar interface deformations.^[^ ^7^, ^8^, ^9^ ^]^ Driven by the capillary interactions, particles assemble into specific structures to reduce the total adsorption free energy. For example, heavy particles tend to form a cluster,^[^ ^11^ ^]^ while ellipsoidal particles assemble into chains.^[^ ^12^, ^13^ ^]^ Furthermore, cubic particles generate hexagonal or honeycomb lattices.^[^ ^14^ ^]^ However, such structures are not dynamically tunable because the capillary interactions are dependent on the intrinsic properties of the particles, such as their weight and their precise shape.

The synthesis of colloidal particles with specific physical properties (e.g., electric or magnetic moments) and anisotropic chemical properties (e.g., amphiphilic Janus particles) interacting with external fields allows for a greater control of the assembly process. For example, magnetic ellipsoids^[^ ^15^, ^16^ ^]^ and magnetic spherical Janus particles^[^ ^17^ ^]^ at fluid interfaces can generate dipolar capillary interactions. Those interactions can be precisely controlled by an external magnetic field. Yet, to the best of our knowledge, so far the assembly of particles was limited to create specific and regular structures, such as chains^[^ ^16^, ^18^ ^]^ or hexagonal lattices.^[^ ^19^ ^]^

Here, we examine a combination of anisotropic physical properties and chemical properties of particles to achieve the formation of diverse structures with controllability and tunablity. We investigate the behavior of magnetic ellipsoidal Janus particles at a flat fluid–fluid interface, interacting with external magnetic fields. We find that by varying its tilt angle due to the presence of an external magnetic field, a single particle generates a dipolar or a hexapolar interface deformation. Driven by the resulting dipolar or hexapolar capillary interactions, such particles assemble into rings, chains, and hexagonal lattice structures, where a dynamical transition between these assembly states can be obtained by dynamically manipulating the field.

We consider an ellipsoidal Janus particle adsorbed at a fluid–fluid interface, as illustrated in **Figure** **1**a,b. The particle is composed of apolar and polar hemispheres of opposite wettability, represented by the three‐phase contact angles θ_A_ = 90° + β and θ_P_ = 90° − β, respectively, where β indicates the amphiphilicity of the particle. The boundary between these two hemispheres is called the Janus boundary. We denote the radii of long‐ and short‐axes of the Janus ellipsoid with *c* and *a*, respectively, and the aspect ratio α is defined as α = *c*/*a*. The magnetic moment *m* is perpendicular to the Janus boundary, and external magnetic fields **H**_*x*_ and **H**_*z*_ are applied in horizontal and vertical direction, respectively. We define magnetic dipole‐field strengths *B_x_ = | **H**_*x*_ || **m*** | and *B_z_ = | **H**_*z*_ || **m*** |, which represent the magnitude of the interactions between the magnetic dipole and the external fields. We apply lattice Boltzmann simulations^[^ ^17^, ^20^, ^21^ ^]^ to investigate the behaviour of magnetic ellipsoidal Janus particles adsorbed at a liquid–liquid interface. A detailed description of the method and simulation parameters is provided in the Supporting Information. In our simulations, magnetic dipole–dipole interaction forces are six orders of magnitude smaller than the capillary interaction forces. Therefore, the dipole–dipole interaction is effectively negligible, and the assembling of structures is purely dominated by the capillary interactions between particles.

**Figure 1:**
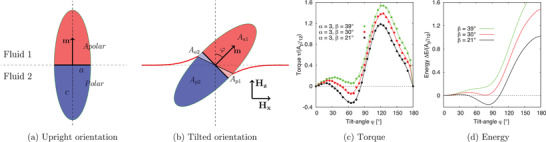
a,b) A single ellipsoidal Janus particle adsorbed at a fluid–fluid interface in an upright orientation (a) and in a tilted orientation (b). c) Reduced torque τ/*A*_p_γ_12_ and d) free energy Δ*E*/*A*_p_γ_12_ of a Janus ellipsoid with aspect ratio α = 3 as a function of tilt angle φ for β = 21° (black) , β = 30° (red) and β = 39° (green), where *A*_p_ = *πac* and γ_12_ is the fluid–fluid interface tension.

In the absence of an external magnetic field, an isolated ellipsoidal Janus particle adsorbed at an interface takes its equilibrium orientation to minimize the total adsorption free energy.^[^ ^22^, ^23^ ^]^ The total adsorption free energy is written as *E* = γ_12_ *A*_12_ + γ_*a*1_ *A*_*a*1_ + γ_*p*2_ *A*_*p*2_ + γ_*a*2_ *A*_*a*2_ + γ_*p*1_ *A*_*p*1_, where γ_*ij*_ are the interface tensions between phases *i* and *j* and *A*_*ij*_ are the contact surface areas between phases *i* and *j*, where *i*, *j* = {1: fluid, 2: fluid, *a*: apolar, *p*: polar}. There is no exact analytical expression for the free energy of a tilted Janus ellipsoid at an interface, due to the difficulty in modeling the shape of the deformed interface and the segment area of the ellipsoid. Our lattice Boltzmann simulations are capable of capturing interface deformations fully without imposing any assumptions about the magnitude of the deformations or stipulating any particle–fluid boundary conditions.^[^ ^17^ ^]^ We take the upright orientation θ = 0 as a reference configuration, and numerically calculate the free energy. To do this, we initialize the particle on the interface with the desired tilt angle and then fix the position and orientation of it. After equlibration of the fluids, we measure the torque subjected on the particles from the fluid–fluid interface in the absence of magnetic fields. We then obtain the free energy Δ*E* = *E*_φ_ − *E*_φ = 0_ by integrating the torque on the particle as the particle rotates quasi‐statically, $\Delta E\;\;=\;\;\int_{0}^{\varphi_{\text{tilt}}}\tau_{\varphi }d\varphi$.

Figure 1c shows the evolution of this torque τ_φ_ versus the tilt angle of a Janus ellipsoid (aspect ratio α = 3) for different amphiphilicities β = 21° (black), β = 30° (red) and β = 39° (green). For all amphiphilicities, the torque is zero at θ = 0° and increases linearly for small tilt angles (φ < 30°), in the direction resisting the rotation of the particle. As the tilt angle increases further φ → 70°, the torque decreases followed by a sharp increase until the tilt angle φ → 130°. Finally, the torque decreases to zero when the tilt angle approaches φ = 180°.

Figure 1d shows the free energy Δ*E* of the ellipsoidal Janus particle, as a function of the tilt angle for the same amphiphilicities as in Figure 1c. For a large amphiphilicity β = 39° the free energy keeps increasing for the whole range of tilt angles, indicating that the particle in the upright orientation φ = 0° corresponds to the global energy minimum. The particle tends to reduce the free energy by increasing the interfacial area between the particle and its preferred fluid phase. For smaller amphiphilicities β = 21° and β = 30°, the free energy is not monotonic: for small tilt angles θ < 30°, the free energy increases, followed by a decrease until the tilt angle increases further to θ ≈ 80°, and afterward, the free energy continuously increases until the tilt angle reaches θ = 180°. For an amphiphility β = 21°, Figure 1d indicates the presence of a local energy minimum for particles in the upright orientation (φ = 0°) and a global energy minimum for particles in the tilted state (φ ≈ 80°). The Janus ellipsoid tends to occupy more of the fluid–fluid surface area and in turn reduces the free energy. An energy barrier exists between the metastable upright orientated configuration and the global energy minimum, which requires a magnetic torque τ_m_ stronger than the capillary torque τ/*A*_p_γ ≈ 0.35 (as shown in Figure 1c), to rotate the particle out of the tilted equilibrium orientation to the upright orientation.

Next, we investigate the interface deformation induced by the ellipsoidal Janus particle at different tilt angles. We find that the interface stays undeformed around the particle with upright orientation, indicating the absence of a torque at φ = 0°, which is consistent with our results in Figure 1c. **Figure** **2** shows how the three‐phase contact line and interface deform around the ellipsoidal Janus particle (α = 3, β = 21°) for different tilt angles φ = 80°, φ = 90° and φ = 120°, respectively. At φ = 80°, the interface deforms around the particle in a hexapolar shape (Figure 2a), with three rises and three dips distributed around the particle. The interface is raised up at the tip of the apolar hemisphere and depressed at the tip of the polar hemisphere. When the particle aligns in the horizontal orientation φ = 90°, the interface shows a dipolar deformation (Figure 2b), with a dip around the apolar hemisphere and a rise around the polar hemisphere. We note that the magnitude of this dipolar deformation is much stronger than that of a hexapolar deformation. Furthermore, the dependence of the mode of deformation on the particle orientation cannot be generated by Janus spheres^[^ ^17^ ^]^ or homogeneous ellipsoids.^[^ ^15^ ^]^ This dependence was also not discovered by previous works on Janus ellipsoids,^[^ ^22^, ^23^ ^]^ where only the equilibrium orientation of Janus ellipsoids were discussed. The deformation increases further with increasing tilt angle to φ = 120° and the deformed interface height is even at the same order of particle short radius (Figure 2c). We also observe an unsymmetrical hexoplar, butterfly‐like deformation at intermediate tilt angles (Figure S1, Supporting Information). The rise and dip generated around the Janus ellipsoid result from the competition between Janus and ellipsoidal properties of the particle: it is known that both, a Janus sphere and a homogeneous ellipsoid, generate dipolar interface deformations. However, the rise and dip areas are located oppositely.^[^ ^15^, ^17^ ^]^ The positioning and strength of the rises and dips can be tuned by varying the aspect ratio and amphiphilicity of the particles, as observed in our simulations (Figure S2, Supporting Information). With increasing either the aspect ratio or the amphiphilicity, the dipolar deformation is expected to dominate for the full range of tilt angles and the hexagonal deformation becomes negligible.

**Figure 2:**
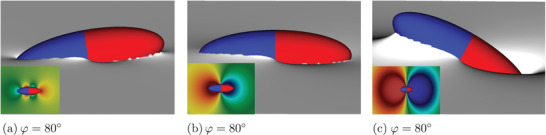
Snapshots of an ellipsoidal Janus particle at a fluid–fluid interface at different tilt angles as obtained from our simulations. The interface deformation appears to be: a) hexapolar at φ = 80° (a), b) dipolar at φ = 90°, and also c) dipolar at φ = 120°, but with a larger deformation. The insets depict the hexapolar/dipolar interface deformation.

If two or more particles are adsorbed at a fluid–fluid interface, the deformations induced by neighboring particles can overlap, leading to capillary interactions between these particles. A pair of Janus ellipsoids interacting through hexapolar or dipolar capillary interactions prefers to align in a side–side configuration (Figure S3, Supporting Information), corresponding to a capillary energy minimum configuration.^[^ ^17^, ^23^ ^]^ However, many‐body effects in the capillary assembling of multiple Janus ellipsoids under external magnetic fields remain to be explored. In **Figure** **3** we show the assembled structures for particle surface fractions Φ = 0.16, 0.62 that form as we vary the dipole‐field strengths *B*_*x*_ and *B*_*z*_. We define the particle surface fraction as Φ = *Nπac*/*A*, where *N* is the total number of particles and *A* is the interface area before particles are placed at the interface. The particles have an aspect ratio α = 3 and an amphiphilicity β = 21°. We define the normalized dipole‐field strength as ${\bar{B}}_{i}=B_{i}/A_{p}\gamma_{12}$, where *i* = *x*, *z*. Initially, the particles are distributed randomly on the interface. In the absence of external magnetic fields, they take their tilted equilibrium orientation φ ≈ 80° and introduce hexapolar interface deformations (as shown in Figure 2a). Then, the particles assemble into locally ordered structures (Figure 3a,f). Small particle clusters with side–side, tip–tip, and side–tip alignments coexist, which indicates that multiple particles interacting through hexapolar capillary interactions have different (meta)stable configurations. When applying a downward magnetic field ${\bar{B}}_{z}=-1.3$, the particles tilt at φ ≈ 140° and generate dipolar interface deformations (as shown in Figure 2c). For a lower particle surface fraction Φ = 0.16, the particles form a circular ring (Figure 3b), instead of a chain structure predicted by the pair‐wise interactions, indicating the presence of strong many‐body interactions. Our results are consistent with the theoretical prediction that a closed loop structure is the capillary energy minimum configuration for multiple tilted ellipsoidal particles interacting with dipolar capillary interactions.^[^ ^24^ ^]^ With increasing the surface fraction Φ = 0.62, the particles form multiple rings and the rings are more curved due to geometrical restriction. Along with ${\bar{B}}_{z}=-1.3$, additionally we apply a horizontal magnetic field ${\bar{B}}_{x}=1.3$. Then, the particles align into chain‐like structures for both lower and higher surface fractions (Figure 3c,h), consistent with the prediction from pair‐wise interactions, demonstrating that many‐body effects are less relevant in this case. We note that both attractive and repulsive capillary forces are present between neighboring particles dependent on their relative orientations. The particles in the same chain touch each other due to attractive forces, whereas, the neighboring chains repel each other, resulting in a clear separation of chains and easy rearrangement of particles with varying external fields. Here, the particles are forced to align in the direction parallel to the horizontal magnetic field and they only have two degrees of freedom (translation in *x* and *y* directions), which weakens the many‐body effect. On the contrary, when only a vertical magnetic field ${\bar{B}}_{z}$ is applied, the particles have three degrees of freedom (translation in *x*, *y* direction and rotation around the *z* axis) and capillary torques can rotate the particles to form rings. If an upward magnetic field ${\bar{B}}_{z}=0.18$ together with a horizontal field ${\bar{B}}_{x}=1.3$ is applied, the particles show a tilt angle φ ≈ 80° and generate hexapolar deformations. For lower particle surface fraction Φ = 0.16, the particles align in a zigzag structure (Figure 3d). At higher surface fraction Φ = 0.64, the particles align in ordered hexagonal lattices (Figure 3i). With only the upward magnetic field ${\bar{B}}_{z}=0.6$ applied, the particles take their upright orientation φ = 0° and align in disordered arrangements due to the absence of capillary interactions (Figure 3e,j). We perform a Voronoi analysis of the structures (Figure S4, Supporting Information), and demonstrate that the disordered structure in Figure 3j has a wider dispersion of Voronoi areas as compared to the locally ordered structure shown in Figure 3f. The assembled structures can be tuned by varying the directions of the magnetic fields (Movie S1, Supporting Information), which has potential applications in sensor or display technology. To estimate the time scale of the assembly and the structural transition, we assume that colloidal particles of radius *a* = 4 µm are adsorbed at a decane‐water interface, with a surface tension γ = 53.2 mN m^−1^, and the effective viscosity μ = 0.91 mPa s.^[^ ^25^, ^26^ ^]^ Based on our simulation results, the estimated time scale of structural formation and transition is about *t* ≈ 1 ms, which is sufficiently fast to satisfy the requirements of responsive materials for advanced sensor or display technologies. For a possible experimental realization of our system, we note that magnetic spherical Janus particles have been experimentally fabricated^[^ ^27^, ^28^, ^29^, ^30^ ^]^ and investigated at a liquid‐liquid interface.^[^ ^25^, ^31^ ^]^ Such Janus particles can have various amphiphilicities^[^ ^32^, ^33^ ^]^ and may be stretched mechanically to form ellipsoidal particles with various aspect ratios.^[^ ^34^, ^35^, ^36^ ^]^ Assuming Janus particles with radius *a* = 100 nm, aspect ratio α = 3 and magnetic moment *m* ≈ 4 × 10^−12^ A m^2^ adsorbed at a water–decane interface with a surface tension $\gamma_{\text{ws}}=70$ mN m^−1^, an external magnetic field *B* ≈ 0.1 T is able to introduce a magnetic torque larger than the capillary torque and to produce the various structures observed in our simulations.

**Figure 3:**
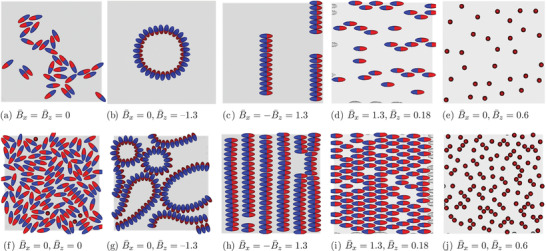
a–j) Assembly of particles with different surface fractions: a–e) Φ = 0.16 and f–j) Φ = 0.62, under different magnetic fields. The particles have an aspect ratio α = 3 and an amphiphilicity β = 21°.

In **Figure** **4** we construct the phase diagram for assembled structures of particles as a function of horizontal ${\bar{B}}_{x}$ and vertical ${\bar{B}}_{z}$ magnetic field‐strengths, showing chains (diamonds), locally ordered clusters (squares), rings (circles), disordered alignments (triangles), and hexagonal lattice structures (pentagons). Locally ordered structures are formed when external magnetic fields are turned off or only a very weak vertical magnetic field $-0.1<{\bar{B}}_{z}<0.3$ is applied. Disordered structures occur when the upward magnetic field is much stronger than the horizontal magnetic field ${\bar{B}}_{z}>1.7{\bar{B}}_{x}$. In this case, the particles take a small tilt angle φ < 30°, where the deformation of the interface is absent or negligible. The particles form hexagonal lattices once a strong horizontal field is applied along with an upward magnetic field satisfying ${\bar{B}}_{x}/{\bar{B}}_{y}>0.6$. Chains are formed when a strong horizontal magnetic field and a downward magnetic field is applied, in the range ${\bar{B}}_{x}/|{\bar{B}}_{z}|>0.4$. The particles assemble into rings when the downward magnetic field is much stronger than the horizontal field $|{\bar{B}}_{z}|/{\bar{B}}_{x}>2.5$.

**Figure 4:**
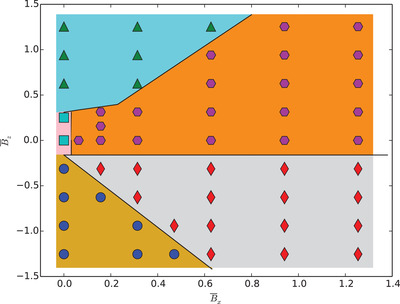
Field‐strength phase diagram of assembled phases of Janus ellipsoids, showing chains (diamonds), locally ordered clusters (squares), rings (circles), disordered alignments (triangles), and hexagonal lattices (pentagons). The particles have an aspect ratio α = 3 and an amphiphilicity β = 21°. The interface fraction covered by particles is Φ = 0.62.

In summary, we demonstrated the controllable self‐assembly of ellipsoidal magnetic Janus particles into clusters, chains, hexagonal lattices and ring‐like structures which can be reconfigured rapidly by applying external magnetic fields. Our results describe a possible way of creating highly ordered and, more importantly, tunable structures at fluid–fluid or nematic–fluid^[^ ^37^ ^]^ interfaces for material assembly. Possible applications are omnipresent not only in the bottom up fabrication of micro/nano‐structured surfaces and materials, but also where the ease of reconfiguration by applying an external magnetic field is of advantage, such as advanced display, sensor, and soft robotics technologies. By applying additionally an oscillating magnetic field, the assembled structures are supposed to propel as colloidal swimmers,^[^ ^38^, ^39^, ^40^, ^41^, ^42^ ^]^ which hold great potential for cargo delivery in biomedical and microfluidic applications.

## Conflict of Interest

The authors declare no conflict of interest.

## Supporting information

Supporting Information

Supporting Movie 1
