# Supplementary material for: Controllable Capillary Assembly of Magnetic Ellipsoidal Janus Particles into Tunable Rings, Chains and Hexagonal Lattices
Source: Adv Mater. 2021 Jan 14;33(8):2006390. doi: 10.1002/adma.202006390 (PMC11468573; doi:10.1002/adma.202006390)
Supplement: Supplementary file 1 — Supporting Information [file ADMA-33-2006390-s002.pdf]

© 2021 Wiley-VCH GmbH

ADVANCED MATERIALS

Supporting Information

for *Adv. Mater.*, DOI: 10.1002/adma.202006390

Controllable Capillary Assembly of Magnetic Ellipsoidal Janus Particles into Tunable Rings, Chains and Hexagonal Lattices

*Qingguang Xie and Jens Harting**

Supplementary Material For "Controllable Capillary Assembly of Magnetic Ellipsoidal Janus Particles into Tunable Rings, Chains and Hexagonal Lattices"

Qingguang Xie[1,*] and Jens Harting[2,3,†]

[1]*Department of Applied Physics, Eindhoven University of Technology, P.O. Box 513, 5600MB Eindhoven, The Netherlands*

[2]*Helmholtz Institute Erlangen-Nürnberg for Renewable Energy (IEK-11), Forschungszentrum Jülich, Fürther Str. 248, 90429 Nürnberg, Germany*

[3]*Department of Chemical and Biological Engineering and Department of Physics, Friedrich-Alexander-Universität Erlangen-Nürnberg, Fürther Straße 248, 90429 Nürnberg, Germany*

[*] q.xie1@tue.nl

[†] j.harting@fz-juelich.de

I. SIMULATION METHOD

We employ the lattice Boltzmann method (LBM) to simulate the motion of each fluid. The method allows for an efficient parallel implementation and has demonstrated itself as a very successful tool for the numerical simulations of fluid flows [1–3]. During almost three decades since its first deployment, the LBM has been improved and extended to allow for the simulation of, for example, multiphase/multicomponent fluids [4–6] and suspensions of particles of arbitrary shape and wettability [7–10].

In the current paper, we use the pseudopotential multicomponent LBM according to the approach of Shan and Chen [4] with a D3Q19 lattice [11] and review some relevant details in the following. Two fluid components follow the Boltzmann equation discretized in space with a lattice constant Δx and in time t with a timestep Δt,

$$f_i^c(\vec{x} + \vec{c}_i \Delta t, t + \Delta t) = f_i^c(\vec{x}, t) + \Omega_i^c(\vec{x}, t), \tag{1}$$

where $i = 1, ..., 19$, $f_i^c(\vec{x}, t)$ are the single-particle distribution functions for fluid component $c = 1$ or 2, $\vec{c}_i$ is the discrete velocity in the ith direction, and

$$\Omega_i^c(\vec{x}, t) = -\frac{f_i^c(\vec{x}, t) - f_i^{\mathrm{eq}}(\rho^c(\vec{x}, t), \vec{u}^c(\vec{x}, t))}{(\tau^c/\Delta t)} \tag{2}$$

is the Bhatnagar-Gross-Krook (BGK) collision operator [12]. τ^c is the relaxation time for component c, while the macroscopic densities and velocities are defined as $\rho^c(\vec{x}, t) = \rho_0 \sum_i f_i^c(\vec{x}, t)$. Here, ρ_0 is a reference density, and $\vec{u}^c(\vec{x}, t) = \sum_i f_i^c(\vec{x}, t)\vec{c}_i/\rho^c(\vec{x}, t)$, respectively. $f_i^{\mathrm{eq}}(\rho^c(\vec{x}, t), \vec{u}^c(\vec{x}, t))$ is a second-order equilibrium distribution function [13]. For convenience we choose the lattice constant Δx, the timestep Δt, the unit mass ρ_0 and the relaxation time τ^c to be unity. This choice leads to a kinematic viscosity $\nu^c = \frac{1}{6}$ in lattice units.

We introduce a repulsive interaction force between fluid components c and c' which effectively mimics a surface tension [4]. Since the pseudopotential LBM of Shan and Chen is a diffuse interface method, this results in an interface width of $\approx 5\Delta x$. The particle is discretized on the fluid lattice and exchanges momentum with the underlying fluid species by means of a modified bounce-back boundary condition as introduced by Ladd and Aidun. [7, 8]. For a detailed description of the method including the extension to particles suspended in multicomponent flows, we refer the reader to some of our recent publications [9, 10, 14–18].

We perform simulations with a system of size $256 \times 128 \times 256$. For single Janus particles, we fix the radii to $a = 10$, $c = 30$. For multiple Janus particles, we reduce the radii to $a = 6, c = 18$. The upper half of the system is filled with fluid 1 and the lower one with fluid 2 leading to a well defined interface in the center of the system. Additionally, we impose walls with mid-grid bounce back boundary conditions at the top and bottom of the system parallel to the interface, while all other boundaries are periodic.

II. SUPPLEMENTARY SIMULATION RESULTS

Supplementary Movie S1 shows the structural transitions when the external magnetic fields are varied.

Fig. S1 shows the interface deformation around a single ellipsoidal Janus particle with aspect ratio $\alpha = 3$ and amphiphilicity $\beta = 21°$ at different tilt angles.

Fig. S2 shows the interface deformation around a single ellipsoidal Janus particle with aspect ratio $\alpha = 3$ and amphiphilicities $\beta = 39°$ at tilt angle $\varphi = 75°$ and $\varphi = 85°$.

Fig. S3 shows the alignment of two ellipsoidal Janus particles with aspect ratio $\alpha = 3$ and amphiphilicity $\beta = 21°$ without and with external magnetic fields.

Fig. S4 shows the Voronoi analysis of the assembled structures of Janus particles with aspect ratio $\alpha = 3$ and amphiphilicity $\beta = 21°$ and a surface coverage fraction $\Phi = 0.64$.

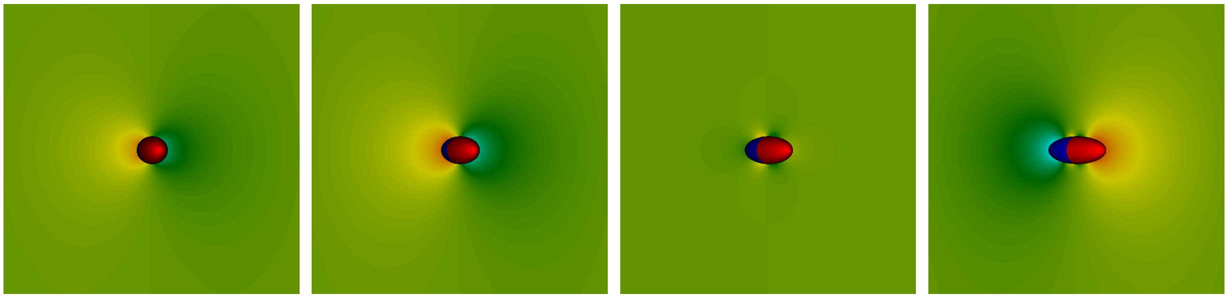

(a) $\varphi = 10°$ (b) $\varphi = 20°$ (c) $\varphi = 30°$ (d) $\varphi = 40°$

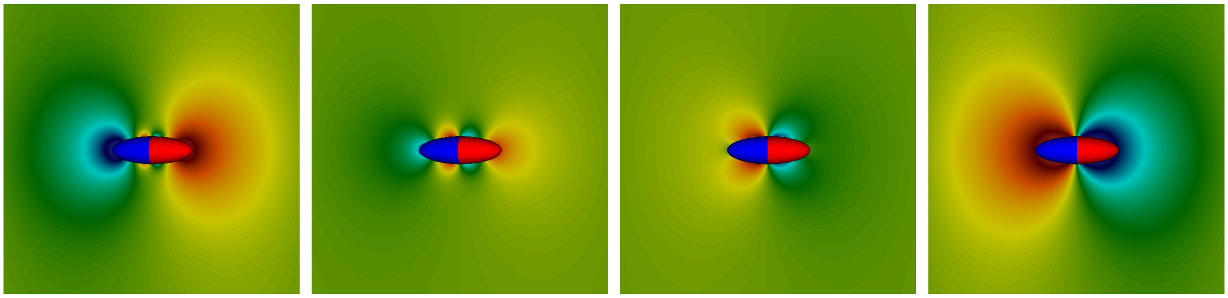

(e) $\varphi = 75°$ (f) $\varphi = 80°$ (g) $\varphi = 85°$ (h) $\varphi = 90°$

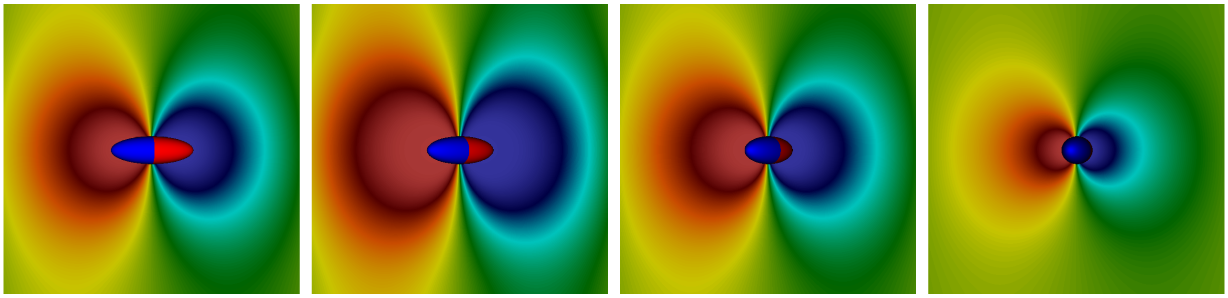

(i) $\varphi = 100°$ (j) $\varphi = 130°$ (k) $\varphi = 150°$ (l) $\varphi = 170°$

FIG. S1: Interface deformation around a single ellipsoidal Janus particle with aspect ratio $\alpha = 3$ and amphiphilicity $\beta = 21°$ at different tilt angles.

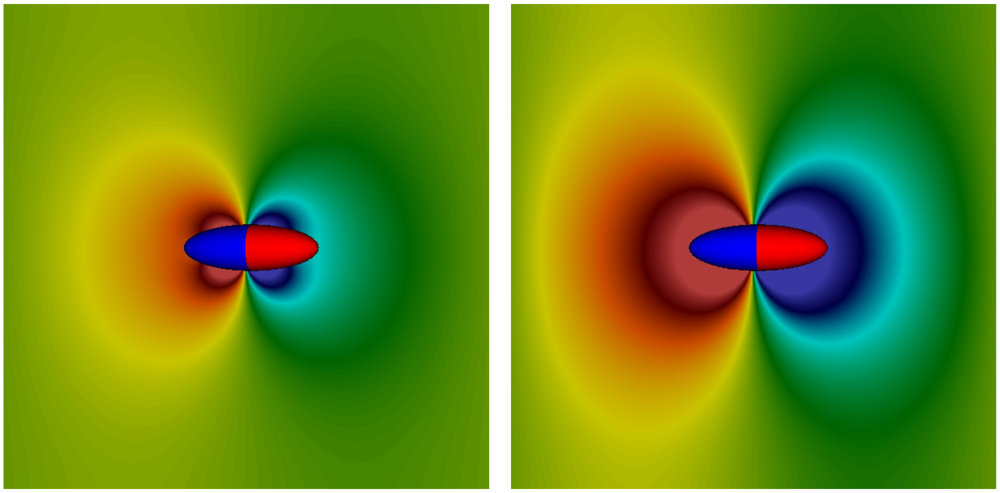

(a) $\varphi = 75°$, $\beta = 39°$ (b) $\varphi = 85°$, $\beta = 39°$

FIG. S2: Interface deformation around a single ellipsoidal Janus particle with aspect ratio $\alpha = 3$ and amphiphilicities $\beta = 39°$ at tilt angle $\varphi = 75°$ and $\varphi = 85°$. The particle generates a unsymmetrical hexapolar interface deformation a) and dipolar deformation b).

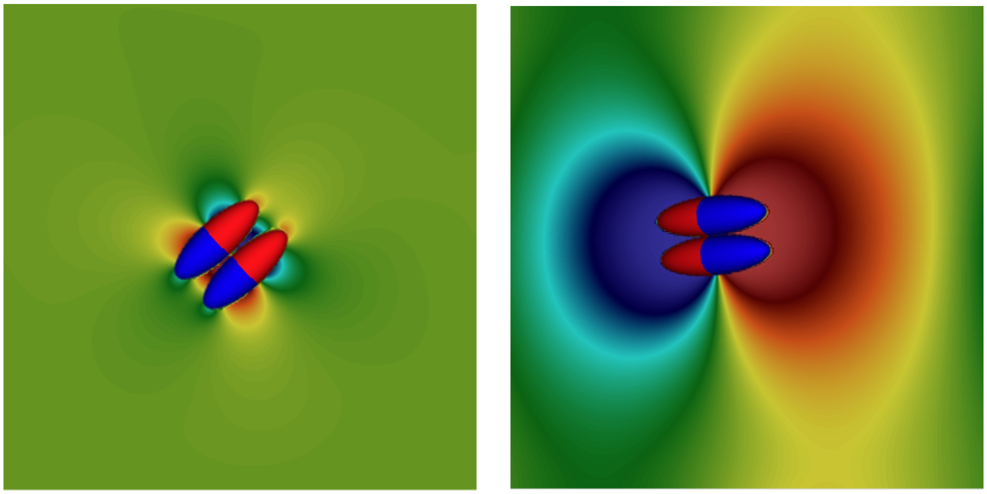

(a) $\bar{B}_x = \bar{B}_z = 0$ (b) $\bar{B}_x = 0$, $\bar{B}_z = -1.2$

FIG. S3: Alignment of two ellipsoidal Janus particle with aspect ratio $\alpha = 3$ and amphiphilicity $\beta = 21°$ without a) and with b) external magnetic fields.

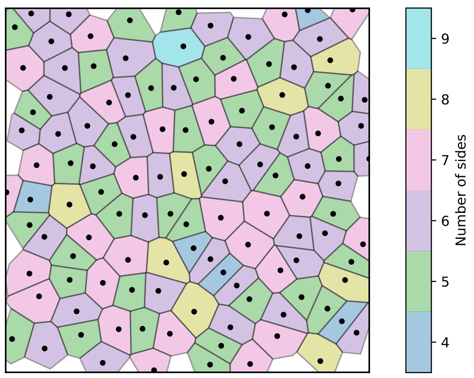

(a) $\bar{B}_x = 0, \bar{B}_z = 0$

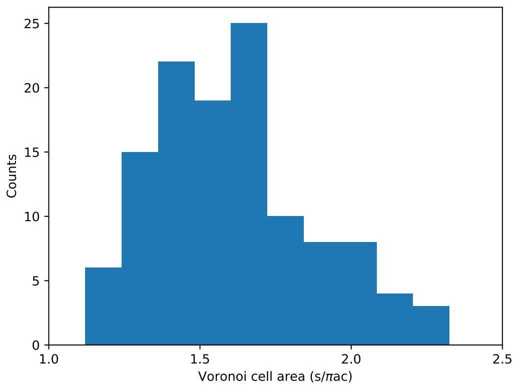

(b) $\bar{B}_x = 0, \bar{B}_z = 0$

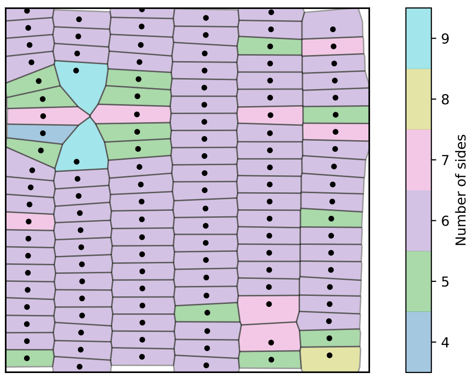

(c) $\bar{B}_x = -\bar{B}_z = 1.3$

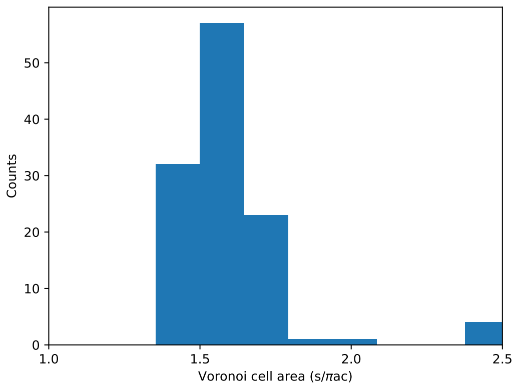

(d) $\bar{B}_x = -\bar{B}_z = 1.3$

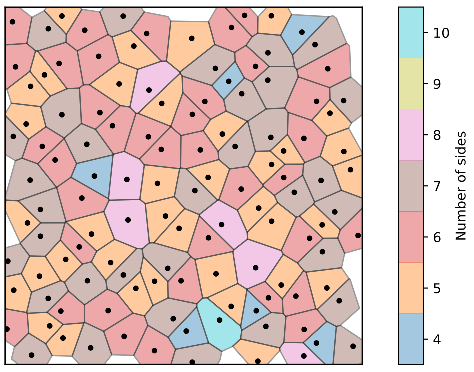

(e) $\bar{B}_x = 0, \bar{B}_z = 0.6$

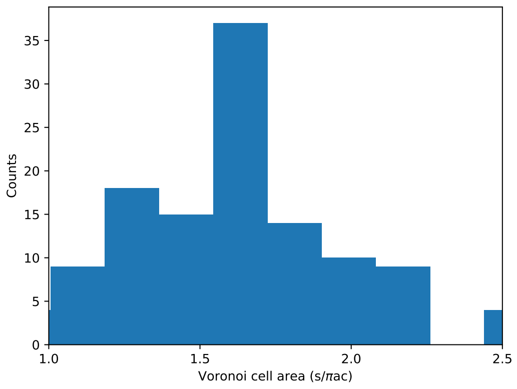

(f) $\bar{B}_x = 0, \bar{B}_z = 0.6$

FIG. S4: Voronoi analysis of structures with surface fraction $\Phi = 0.62$ under different magnetic fields. The disordered structure $\bar{B}_x = 0, \bar{B}_z = 0.6$ has a wider dispersion of Voronoi areas as compared to the locally ordered $\bar{B}_x = 0, \bar{B}_z = 0$ and chain-like $\bar{B}_x = -\bar{B}_z = 1.3$ structures.

[1] R. Benzi, S. Succi, and M. Vergassola. The lattice Boltzmann equation: theory and applications. *Phys. Rep.*, 222:145, 1992.

[2] S. Succi. *The Lattice Boltzmann Equation: For Fluid Dynamics and Beyond.* Oxford University Press, 2001.

[3] T. Krüger, H. Kusumaatmaja, A. Kuzmin, O. Shardt, G. Silva, and E.M. Viggen. *The Lattice Boltzmann Method - Principles and Practice.* Springer, 2016.

[4] X. Shan and H. Chen. Lattice Boltzmann model for simulating flows with multiple phases and components. *Phys. Rev. E*, 47:1815, 1993.

[5] X. Shan and H. Chen. Simulation of nonideal gases and liquid-gas phase transitions by the lattice Boltzmann equation. *Phys. Rev. E*, 49:2941, 1994.

[6] H. Liu, Q. Kang, C. R. Leonardi, S. Schmieschek, A.l Narváez, B. D. Jones, J. R. Williams, A. J. Valocchi, and J. Harting. Multiphase lattice Boltzmann simulations for porous media applications. *Computat. Geosci.*, 20:777–805, 2016.

[7] A. J. C. Ladd and R. Verberg. Lattice-Boltzmann Simulations of Particle-Fluid Suspensions. *J. Stat. Phys.*, 104:1191–1251, 2001.

[8] C. K. Aidun, Y. Lu, and E.-J. Ding. Direct Analysis of Particulate Suspensions with Inertia Using the Discrete Boltzmann Equation. *J. Fluid Mech.*, 373:287–311, 1998.

[9] F. Jansen and J. Harting. From bijels to Pickering emulsions: a lattice Boltzmann study. *Phys. Rev. E*, 83:046707, 2011.

[10] F. Günther, F. Janoschek, S. Frijters, and J. Harting. Lattice Boltzmann Simulations of Anisotropic Particles at Liquid Interfaces. *Comput. Fluids*, 80:184–189, 2013.

[11] Y. H. Qian, D. D'Humières, and P. Lallemand. Lattice BGK models for Navier-Stokes equation. *Europhys. Lett.*, 17:479–484, 1992.

[12] P.L. Bhatnagar, E.P. Gross, and M. Krook. A model for collision processes in gases. I. Small

amplitude processes in charged and neutral one-component systems. *Phys. Rev.*, 94:511–525, 1954.

[13] H. Chen, S. Chen, and W. H. Matthaeus. Recovery of the Navier-Stokes equations using a lattice-gas Boltzmann method. *Phys. Rev. A*, 45, 1992.

[14] A. Komnik, J. Harting, and H. J. Herrmann. Transport phenomena and structuring in shear flow of suspensions near solid walls. *Journal of Statistical Mechanics: theory and experiment*, P12003, 2004.

[15] S. Frijters, F. Günther, and J. Harting. Effects of nanoparticles and surfactant on droplets in shear flow. *Soft Matter*, 8:6542–6556, 2012.

[16] S. Cappelli, Q. Xie, J. Harting, A. M. de Jong, and M. W. J. Prins. Dynamic wetting: status and prospective of single particle based experiments and simulations. *New Biotechnol.*, 32:420–32, 2015.

[17] T. Krüger, S. Frijters, F. Guñther, B. Kaoui, and J. Harting. Numerical simulations of complex fluid-fluid interface dynamics. *Eur. Phys. J. Special Topics*, 222:177–198, 2013.

[18] Q. Xie and J. Harting. From dot to ring: the role of friction on the deposition pattern of a drying colloidal suspension droplet. *Langmuir*, 34:5303–5311, 2018.

[19] W. Fei, M. M. Driscoll, P. M. Chaikin, and K. J.M. Bishop. Magneto-capillary dynamics of amphiphilic Janus particles at curved liquid interfaces. *Soft Matter*, 14:4661–4665, 2018.

[20] W. Fei, P. M. Tzelios, and K. J. M. Bishop. Magneto-capillary particle dynamics at curved interfaces: Time-varying fields and drop mixing. *Langmuir*, 36:6977–6983, 2020.

[21] I. Sinn, P. Kinnunen, S. N. Pei, R. Clarke, B. H. McNaughton, and R. Kopelman. Magnetically uniform and tunable Janus particles. *Appl. Phys. Lett.*, 98:024101, 2011.

[22] S. K. Smoukov, S. Gangwal, M. Marquez, and O. D. Velev. Reconfigurable responsive structures assembled from magnetic Janus particles. *Soft Matter*, 5:1285, 2009.
